# Whole-genome resequencing reveals genetic differences and the genetic basis of parapodium number in Russian and Chinese *Apostichopus japonicus*

**DOI:** 10.1186/s12864-023-09113-x

**Published:** 2023-01-16

**Authors:** Chao Guo, Xianglei Zhang, Yuanxin Li, Jiahui Xie, Pingping Gao, Pengfei Hao, Lingshu Han, Jinyuan Zhang, Wenpei Wang, Peng Liu, Jun Ding, Yaqing Chang

**Affiliations:** 1 Key Laboratory of Mariculture & Stock Enhancement in North China’s Sea, Ministry of Agriculture and Rural Affairs, Dalian Ocean University, Dalian, Liaoning 116023 People’s Republic of China; 2 Ningbo University, Ningbo, Zhejiang 315211 People’s Republic of China

**Keywords:** *Apostichopus japonicus*, Resequencing, Genome-wide association analysis, Population structure, Population genetics

## Abstract

**Background:**

*Apostichopus japonicus* is an economically important species in the global aquaculture industry. Russian *A. japonicus*, mainly harvested in the Vladivostok region, exhibits significant phenotypic differentiation, including in many economically important traits, compared with Chinese *A. japonicus* owing to differences in their habitat. However, both the genetic basis for the phenotypic divergence and the population genetic structure of Russian and Chinese *A. japonicus* are unknown.

**Result:**

In this study, 210 individuals from seven Russian and Chinese *A. japonicus* populations were sampled for whole-genome resequencing. The genetic structure analysis differentiated the Russian and Chinese *A. japonicus* into two groups. Population genetic analyses indicated that the Russian population showed a high degree of allelic linkage and had undergone stronger positive selection compared with the Chinese populations. Gene ontology terms enriched among candidate genes with group selection analysis were mainly involved in immunity, such as inflammatory response, antimicrobial peptides, humoral immunity, and apoptosis. Genome-wide association analysis yielded eight single-nucleotide polymorphism loci significantly associated with parapodium number, and these loci are located in regions with a high degree of genomic differentiation between the Chinese and Russia populations. These SNPs were associated with five genes. Gene expression validation revealed that three of these genes were significantly differentially expressed in individuals differing in parapodium number. AJAP08772 and AJAP08773 may directly affect parapodium production by promoting endothelial cell proliferation and metabolism, whereas AJAP07248 indirectly affects parapodium production by participating in immune responses.

**Conclusions:**

This study, we performed population genetic structure and GWAS analysis on Chinese and Russian *A. japonicus*, and found three candidate genes related to the number of parapodium. The results provide an in-depth understanding of the differences in the genetic structure of *A. japonicus* populations in China and Russia, and provide important information for subsequent genetic analysis and breeding of this species.

**Supplementary Information:**

The online version contains supplementary material available at 10.1186/s12864-023-09113-x.

## Background

*Apostichopus japonicus* (Echinodermata, Holothuroidea) is mainly distributed along the North Pacific coast, including Russia, Japan, Korea, and the northern coast of China. *A. japonicus* possesses a range of pharmacological activities, including anti-inflammatory, antioxidant, and antitumor effects, which imparts it with a high medicinal value [1]. These effects are attributed to its bioactive compounds such as polysaccharides, proteins, and cytokines, as well as antioxidant compounds like vitamin E and C, and carotenoids [2, 3]. In recent years, with the increase in the value and demand for *A. japonicus*, there has been a trend of significant growth in *A. japonicus* aquaculture production [4]. According to data from the 2021 China Fishery Statistical Yearbook, China's *A. japonicus* production reached 196,564 tons.

The external morphology of *A. japonicus* varies between geographic populations, with Russian individuals having a greater number of parapodium and thicker walls than Chinese individuals [5]. The parapodium of *A. japonicus* are found on the back and sides, which is another critical factor in measuring their market value apart from their body colour [5]. The tip of the parapodium of the *A. japonicus* is rich in nerve plexus cells and sensory cells [6], which play an essential role in the life activities of the *A. japonicus* as a sensory organ [7]. In addition, it has been shown that histamine signaling molecules are present in the ciliated cells of the *A. japonicus* parapodium and are involved in signaling in the epidermal layer of the parapodium [8].

Sea cucumber farming in China has a long history, dating back to the Shang Dynasty, 1300 B.C., and has undergone a long period of domestication and extensive selection [9]. However, conventional domestication methods have a long breeding cycle and several generations are usually required to select for favorable and stable traits [10]. Using next-generation sequencing to analyze the genomic structure of a species can provide insights into the genetic diversity of the species and improve breeding efficiency [11]. Genome-wide association studies (GWAS) enables simultaneous detection of multiple alleles (suitable for localization of quantitative traits) in populations [12], and can be used to identify loci or gene associated with important agronomic traits [13]. To date, several studies have used GWAS to identify SNP locations associated with *A. japonicus* phenotypes. For example, GWAS were applied by Wang et al [14]. to identify loci associated with *A. japonicus* sex determination, which were found to be distributed on Chr4, Chr9, Chr17, and Chr18. This confirmed the possibility of an XX/XY system of sex determination in sea cucumbers. Using GWAS, Ge et al [15]. identified 10 SNP-associated loci related to *A. japonicus* color variation, located on Chr17, Chr21, Chr15, and Chr05, and determined that endothelin-converting enzyme-1 may be a key enzyme in the occurrence of color variation. Additionally, in our previous research, GWAS was used to identify potential SNP-associated loci for *A. japonicus* traits such as the number of parapodium and growth traits [14, 16]. Furthermore, GWAS also has been widely used to systematically screen loci or genes associated with economically important traits in others aquatic animals, such as fish [17], shellfish [18], and crustaceans [19].

To identify loci or genes associated with parapodium numbers of *A. japonicus* and to facilitate targeted and precise genetic selection involving marker-assisted selection (MAS) and molecular design breeding, a total of 210 samples from seven populations of *A. japonicus* in Russia and China were whole-genome re-sequenced using the Illumina NovaSeq 6000 sequencing platform. Unlike our previous research on the number of parapodium in *A. japonicus*, this study introduced a population of Russian *A. japonicus* with a high number of parapodium, increasing the richness of phenotypic variation and aiding in the identification of more reliable SNP-associated loci.

## Materials and methods

### Sample collection and trait measurement

**Fig. 1:**
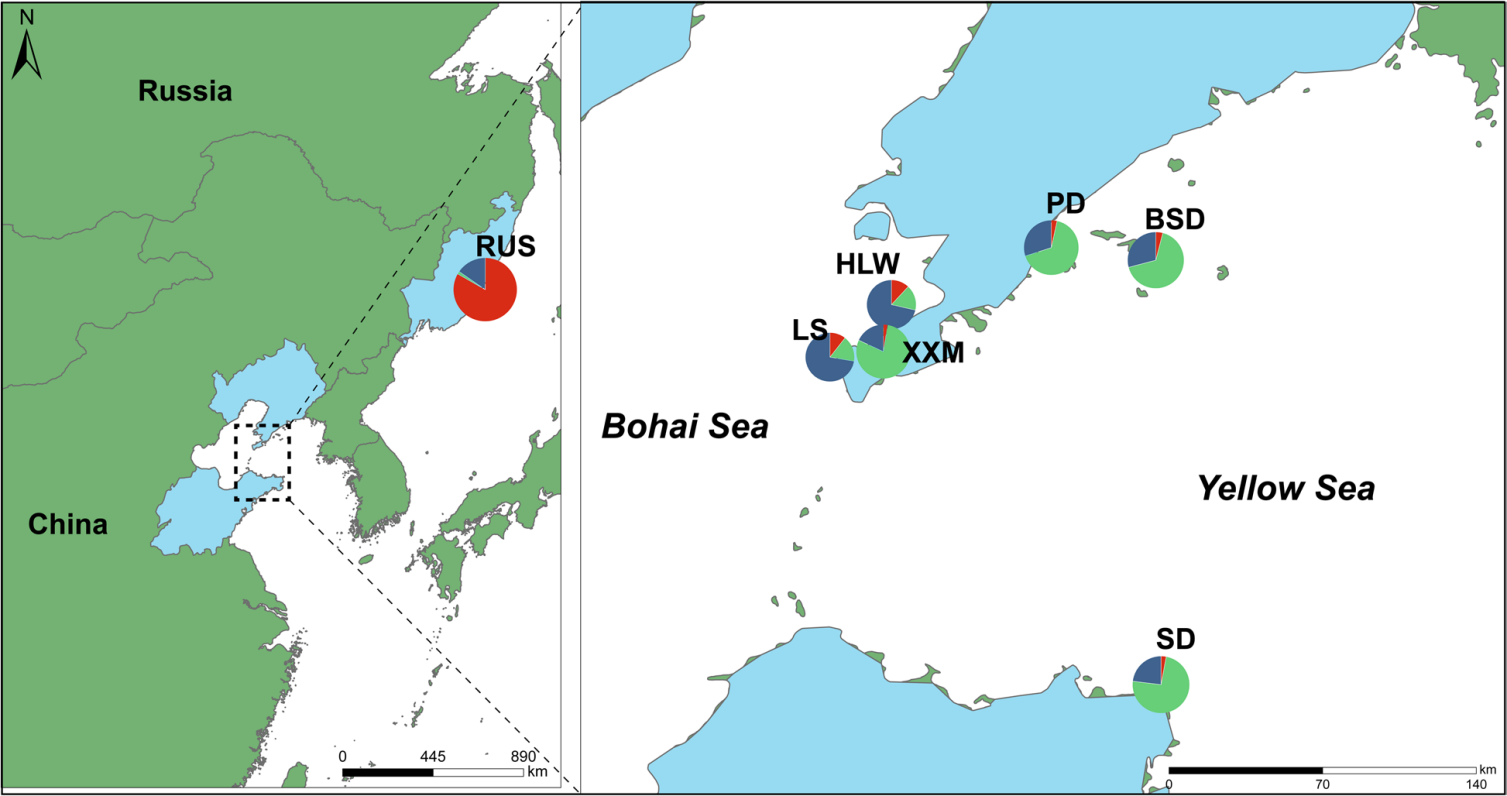
The sampling location of the A. japonicus: PingDao (PD), XiXiaoMo (XXM), BaShaoDdao (BSD), ShanDong (SD), HuangLongWei (HLW), LvShun (LS), and Russian A. japonicus (Vladivostok, *n* = 30). The pie chart represents the population structure of the sampling location

*Apostichopus japonicus* populations from the Yellow Sea and the Bohai Sea regions were selected: PingDao (PD, Liaoning Province, Yellow Sea coast, *n* = 30), XiXiaoMo (XXM, Liaoning Province, Yellow Sea coast, *n* = 30), BaShaoDdao (BSD, Liaoning Province, Yellow Sea coast, *n* = 30), ShanDong (SD, Shandong Province, Yellow Sea coast, *n* = 30), HuangLongWei (HLW, Liaoning Province, Bohai Sea coast, *n* = 30), LvShun (LS, Liaoning Province, Bohai Sea coast, *n* = 30), and Russian *A. japonicus* (Vladivostok, *n* = 30), with sample collection sites shown in Fig. 1.

The *A. japonicus* were collected from July to December 2020 and transferred to the Key Laboratory of Northern Seawater Aquaculture of the Ministry of Agriculture in rural areas for 3 weeks, during which time the water was kept clear and turbidity-free and fed with seaweed slurry with Spirulina until sampling was completed. Three 1 cm × 1 cm samples of muscle tissue were taken from healthy *A. japonicus*, frozen in liquid nitrogen immediately after sampling and stored in 2 ml centrifuge tubes at -80 °C.

**Fig. 2:**
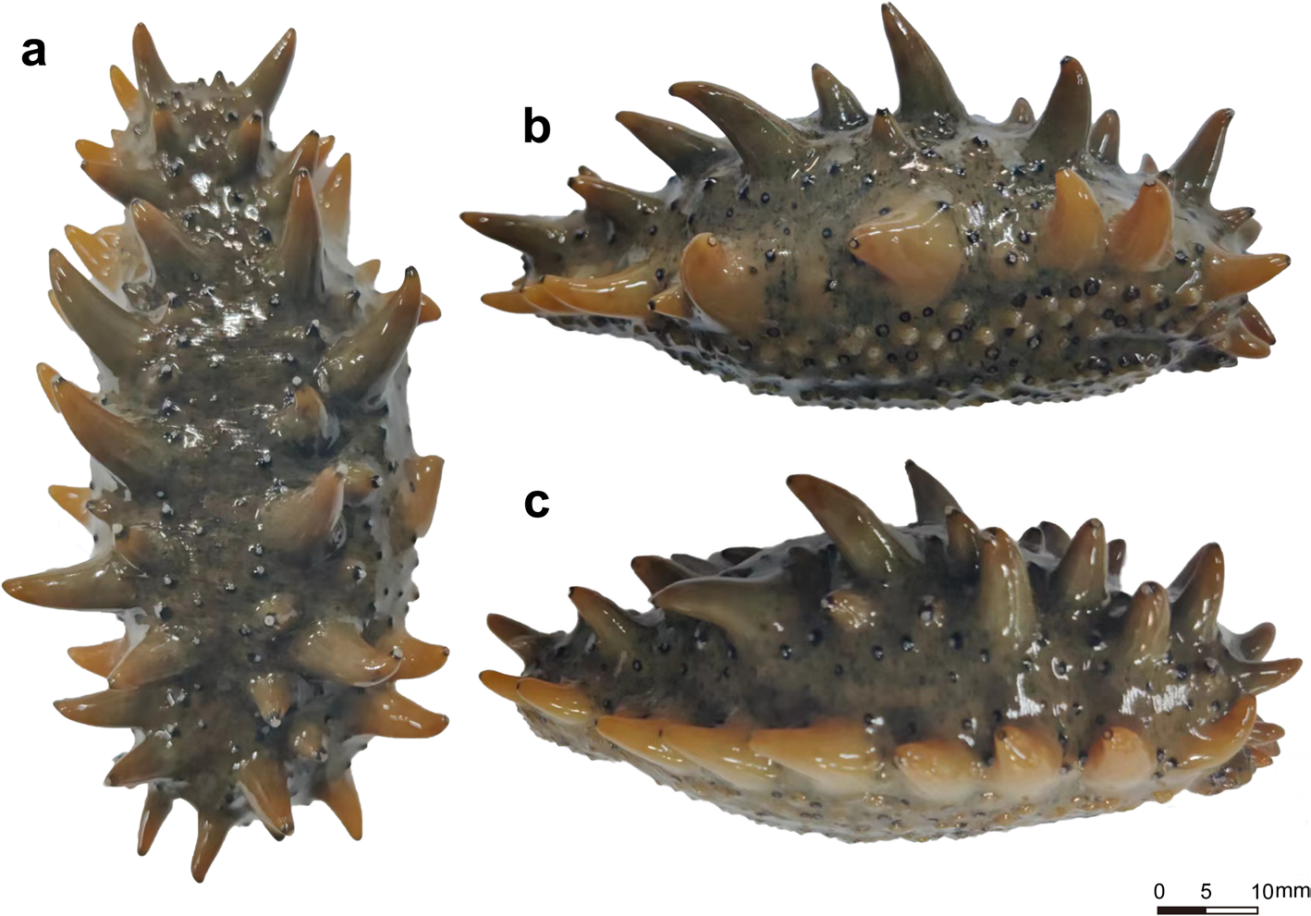
*A. japonicus* parapodium Note: **a**: back. **b**: left. **c**: right

Healthy *A. japonicus* with well-developed parapodium (Fig. 2) were selected for this experiment and the number of parapodium was counted using the method established by Chang et al. [5], and measurements were averaged by repeating the counts three times. The number of parapodium was tested for normality using the R language " Kolmogorov–Smirnov test ", and the density of trait distribution for the total sample was plotted using the R package ggplot2.

### Total DNA extraction and genome resequencing

The TIANGEN amp Marine Animals DNA Kit (TIANGEN, Beijing, China) was used for the extraction of total genomic DNA in this experiment. *A. japonicus* muscle was selected instead of the body wall tissue, to avoid the influence of complex organic substances in the body wall, which might affect the quality of nucleic acids. The purity and integrity of the nucleic acids were initially checked by 1% agarose gel electrophoresis, and samples with clear electrophoretic bands and no or mild degradation were qualified. Qualified DNA samples were adjusted to 100 ng/μl and stored at -20 °C.

Qualified DNA samples were randomly fragmented into 350 bp fragments using a Covaris E220 ultrasonic DNA fragmentation machine (Shanghai Tusheng Vision Technology Co., Ltd., Shanghai, China). The library preparation process was completed on a T100 Thermal Cycler PCR (BIO-RAD, USA) instrument using KAPA HyperHlus reagent (Illumina, USA) after 10 cycles of end repair, addition of polyA tails, addition of sequencing adapters, and purification and amplification. High-throughput sequencing of samples was performed using the Illumina Nova6000 sequencing platform (Illumina, California, USA) with a 150 bp paired-end reads (LC-Bio, Hangzhou, Zhejiang Province, China).

### SNP calling

High-quality trimmed reads were compared with consensus sequences obtained by clustering with BWA software [20] reads and with the whole genome of the *A. japonicus* (https://ftp.ncbi.nlm.nih.gov/genomes/all/GCA/002/754/855/GCA_002754855.1_ASM275485v1/) for comparison. The reference genome we selected is the one with the longest N50 length among the published *A. japonicus* genomes, with a contig N50 of 190 Kb and a scaffold N50 of 486 Kb [21]. Single Nucleotide Polymorphism (SNP) were detected on GATK software [22], and the quality of variation sites was filtered to obtain possible SNP information of the sample. According to QD < 2.0, MQ < 40.0, FS > 60.0, MQRankSum < -12.5 and ReadPosRankSum < -8.0, the SNPs with two-allele polymorphism were retained, and SNPeff [23] was used to annotate the mutation sites.

### Estimates of heritability for traits in the number of parapodium

Heritability estimates for traits in the number of parapodium in the *A. japonicus* were performed using GCTA software (v1.93.2) based on SNP data using the EM-REML approach [24]. Since SNPs may have a high degree of linkage disequilibrium, which may reduce the accuracy of estimating genetic parameters for individual animals, this study used the Average Euclidean distance (AED) of SNP gene frequencies to screen SNPs and selected SNPs with 50 K density for heritability estimation.

### Population structure and phylogenetic analysis

To identify independent SNPs for phylogenetic tree construction and structural analyses, LD coefficients (r^2^) were calculated using a sliding window of 100 kb with a sliding step of 10 kb, and a subset of 585,778 SNPs were extracted from the SNP dataset of all samples using an LD threshold of r^2^ < 0.05. Population structure analyses were performed in Admixture [25] (v1.3.0) with *K* values set from 1 to 9. Phylogenetic tree analyses were performed using VCF2Dis (https://github.com/BGI-shenzhen/VCF2Dis) to construct nj trees.

### PCA and LD decay

Data for PCA analysis were similarly analysed using independent SNPs (the subset of 585,778 SNPs), and PCA analysis was performed using Plink [26] (v1.90b6.21) software to analyse values corresponding to PC1 to PC10, and PCA plots were drawn using the R language ggplot2 package. LD decay was performed using data unfiltered by r^2^ < 0.05, using PopLDdecay [27] software (v3.41) for analysis.

### Population selection analysis

Population selection analyses were performed using vcftools [28] (v0.1.16) using a 100 kb sliding window with 10 kb sliding steps to calculate inter-population *F*_*ST*_ analyses and intra-population nucleotide diversity (π). Fixation index (*F*_*ST*_) analysis and reduction of diversity (ROD) analysis were used to identify regions of high divergence at the genomic level in Russian and Chinese *A. japonicus*. The *F*_*ST*_ values between the two groups were calculated using a 100 kb sliding window (sliding step of 10 kb), and the sliding window with the top 5% of *F*_*ST*_ values was selected as a highly divergent candidate region. Tajima's D test within populations was performed separately using vcftools using a 100 kb non-overlapping sliding window. A perl script was used based on $\mathrm{ROD}=1-\frac{Pi pop1}{Pi pop2}$ (*Pipop1* and *Pipop2* are the values of nucleotide diversity (π) of the two populations) Calculation of inter-population ROD values [29].

### Gene ontology analysis

Using the eggNOG-mapper [30] (v5.0), homology annotation was performed based on the genomic protein sequence files of the *A. japonicus* to obtain correspondence between genes and GO terms, the OrgDb package for GO analysis were constructed using the R package AnnotationForge [31], and enrichment analysis was performed using the R package clusterProfile (v4.2.0) [32].

### Genome-wide association analysis and candidate gene identification for the number of parapodium

Genome-wide association analysis was performed using a mixed linear model (LMM) in the GEMMA [33] (0.98.3) software. As the parapodium number trait in spiny cucumbers is a high heritability trait, we ignored the effect of environmental effects on the phenotype and added the phenotypic values of parapodium number directly to the GWAS analysis.

Based on the Bonferroni correction method according to $p=\frac{0.05}{Number of SNPs}$ The screening threshold was calculated and the significance screening threshold for relevant SNPs was -log_10_ *P* > 7.4. For the significant SNP loci obtained, the following strategy was used for analysis: firstly, the corresponding genes and associated functions were found in the spiked genome annotation file based on the SNP location information. Secondly, to analyse the functions of these candidate genes more accurately, eggNOG was used to annotate and find their cognate gene functions. Thirdly, these SNPs were analysed for upstream and downstream genomic LD blocks. Fourthly to explore whether the significant SNP loci were located within the selected genomic regions obtained from *F*_*ST*_ and ROD analysis.

### qRT-PCR for gene expression analysis

qRT-PCR was used for gene expression analysis. Primers were designed by Primer 5.0 (Supplementary table 1) and five genes associated with genes related to the parapodium number trait were selected for qRT-PCR detection. Cytochrome b (cytb) gene was used as an internal reference gene. Sample validation for the number of spines selected parapodium tissue with spine numbers of 33 (few parapodium), 40 (control) and 55 (more parapodium), with three parallel samples per group. Total RNA was extracted using the RNeasy kit (Tiangen), and tested for purity (A260/280 ratios) and integrity (denaturating gel electrophoresis). For qRT-PCR, the RNA was converted to cDNA using the High-Capacity cDNA reverse transcription kit (Tiangen). Gene expression levels were calculated by the 2^−ΔΔCt^ method. The Student's t-test was used to assess the difference between groups.

## Results

### High-throughput sequencing results

210 *A. japonicus* samples were sequenced using an Illumina high-throughput sequencing platform. An average of 78,153,766.5 (total 11.72 × 10^9^) raw reads were obtained per sample. After removal of low-quality sequences and adaptor sequences, 2180.6 × 10^9^ valid reads were retained with an average valid read rate of 86.12% (Table 1). The GC content was 37.45%-40.02%, the Q20 was 92.05%-97.46%, and the Q30 was 85.42%-93.39% for an individual sample (Table 1). The mean number of SNPs per sample was 8,852,668.30 (Table 1). Genotyping of the SNP loci revealed slight differences in the proportion of genotypic heterozygosity between populations; the highest heterozygosity (0.23) was detected in the HLW population and the lowest heterozygosity (0.20) was observed in the SD population (Table 1). The SNPs were unevenly distributed across the genome, with 66.78% located between genes, 21.86% in introns, 4.15% in exons, and 3.42% and 3.48% upstream and downstream of genes, respectively (Supplementary Fig. 1). The final total of 6,836,886 high-quality SNP loci met the requirements for subsequent analysis by filtering (criteria: minor allele frequency < 0.05, maximal proportion of missing data 0.8).

**Table 1:** Whole genome resequencing results of *Apostichopus japonicus*

Groups	Reads mapping rate %	Q20%	Q30%	GC%	Average sequencing depth	Number of SNPs	The proportion of heterozygous loci%
BSD	81.29	94.16	88.04	37.45	10.77	9,141,887.40	0.22
PD	86.23	95.75	90.75	37.89	11.98	8,941,680.23	0.21
HLW	89.36	95.49	90.92	39.82	12.44	9,211,083.43	0.23
SD	80.89	94.62	88.95	37.70	11.25	8,808,006.8	0.20
XXM	87.07	96.04	91.16	37.74	13.03	8,730,540.47	0.22
LS	90.28	95.84	91.39	39.55	12.22	9,059,672.47	0.21
RUS	88.40	95.41	90.82	40.02	12.41	9,173,089.17	0.22

### Phenotypic statistics for the number of parapodium and its heritability

Statistical analysis of the number of parapodium of the seven *A. japonicus* populations (Table 2) showed that the Russia group had the highest number of parapodium (mean 56.67). The HLW and LS populations had relatively low numbers of parapodium (means of 34.63 and 34.23, respectively). The number of parapodium in all seven populations was normally distributed (Table 2) and the parapodium number in a total of 210 samples was approximately normally distributed (Supplementary Fig. 2). Heritability can reflect the proportion of variation in a population for a trait that is not explained by environment or random chance [34]. The heritability of the number of parapodium in *A. japonicus*, calculated with the GCTA software based on the SNP data, was 0.81–0.91, thus the trait showed high heritability [35] (Table 2). Based on this result in the GWAS analysis, we ignored the effect of environmental factors on the number of parapodium and directly used the phenotypic value for subsequent analysis.

**Table 2:** Statistical results of parapodium number phenotype and heritability of *A. japonicus*

Groups	Parapodium number	P	h^2^
BSD	47.90 ± 9.29	0.55	0.90
PD	44.47 ± 5.95	0.94	0.86
HLW	34.63 ± 4.71	0.56	0.83
SD	40.80 ± 4.32	0.73	0.81
XXM	50.50 ± 9.03	0.78	0.90
LS	34.23 ± 5.05	0.65	0.84
RUS	56.67 ± 10.54	0.96	0.91

*P represents the significance of normal distribution

h^2^ represents the heritability of parapodium number

### Analysis of population genetic structure and genetic differentiation

**Fig. 3:**
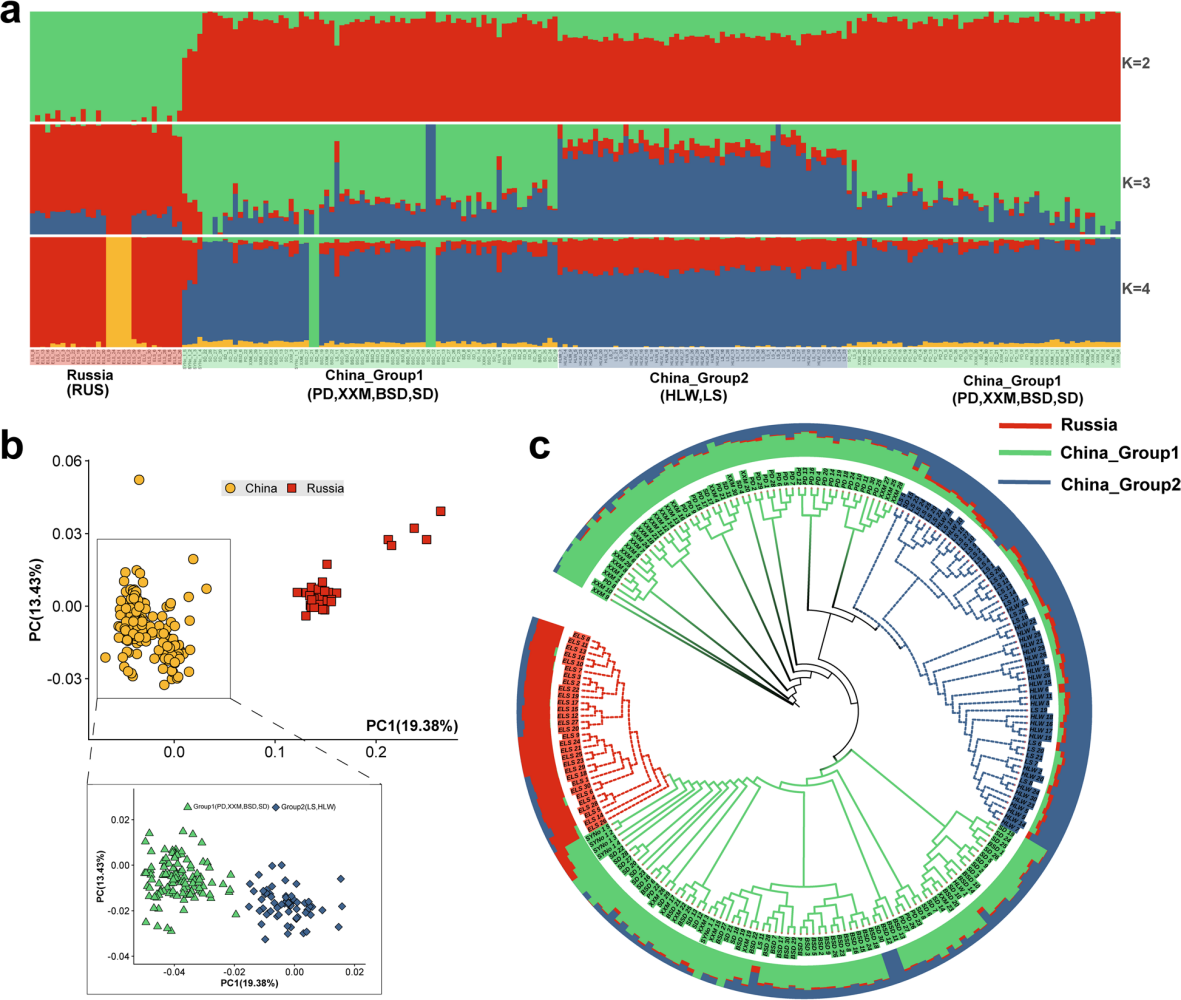
Population genetic structure analysis of *A. japonicas* Note: **a**: Population structure of *A. japonicus*, each column represents an individual, and the individual is matched with the phylogenetic tree branches in Figure c in turn, the red block represents the Russia subgroup, the green block represents the China_Group1(PD, XXM, BSD, SD) subgroup, and the blue block represents the China_Group2 (HLW, LS) subgroup. **b**: PCA figure. **c**: Phylogenetic tree

Admixture population structure analysis showed that the structure of the Russia population differed from that of the other six populations of *A. japonicus* at *K* = 2, 3, and 4. We divided the RUS populations into one group and the PD, XXM, BSD, SD, HLW, LS populations into another group, namely the Russia and China groups, based on their geographical characteristics. It was noteworthy that the HLW and LS populations within the China group showed a different group structure from the other four China populations at *K* = 3 or 4. Therefore, we further divided the China group into two subgroups, China_Group1 and China_Group2 (Fig. 3a).

A principal component analysis (PCA) was conducted in which the proportion of the total variance explained by PC1 and PC2 was 19.38% and 13.43%, respectively. The PCA plots (Fig. 3b) showed that PC1 separated the China group from the Russia group, and further analysis of the China group revealed that PC1 distinguished the HLW and LS populations from the other four Chinese populations into two subgroups, which was consistent with the results of the population structure analysis. Phylogenetic tree reconstruction showed that the Russia group formed a distinct cluster and the China group showed two clustering (Fig. 3c): the HLW and LS populations were more closely related and the other four populations showed separate clustering of individuals rather than of populations.

**Fig. 4:**
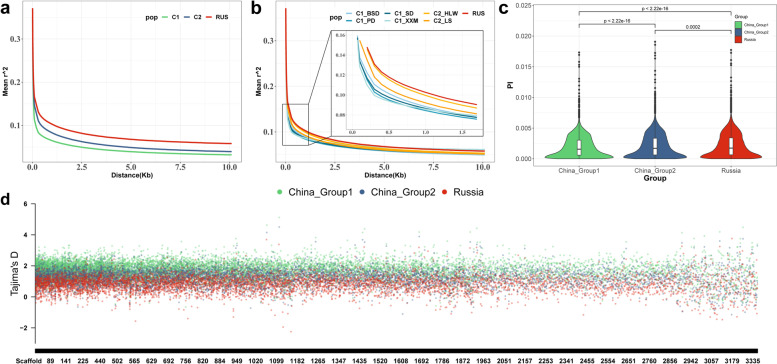
Genetic differentiation among *A. japonicus* subpopulations Note: **a** Linkage disequilibrium for three subgroups. **b** Linkage disequilibrium for seven sampling groups. **c** Nucleotide diversity analysis using 100 kb sliding windows and Wilcoxon test for significance testing. **d** Nucleotide polymorphisms (Pi) in three subpopulations. The red block represents the Russia subgroup, the green block represents the China_Group1 subgroup, and the blue block represents the China_Group2 subgroup

The physical distance corresponding to a LD coefficient *r*^2^ = 1 was used as the LD attenuation distance. The LD analysis showed that, among the different subgroups, the Russia group had the longest LD decay distance (~ 1.2 kb), China_Group2 had the second longest LD decay distance (~ 0.5 kb), and China_Group1 had the shortest LD decay distance (~ 0.25 kb) (Fig. 4a). For the sampled populations, the Russia group had the longest LD decay distance, the HLW and LS populations in China_Group2 had the second longest LD decay distance, and the XXM population in China_Group1 had the shortest LD decay distance (Fig. 4b). These results suggested that the allele of Russian *A. japonicus* group exhibited a higher degree of inter-locus linkage and experienced more strongly positive selection compared with the Chinese *A. japonicus* group.

Nucleotide diversity (π) intuitively reflects the genetic diversity of the population. The results of the nucleotide diversity analysis for the three subgroups are shown in Fig. 4c. The π values for China_Group1 were significantly lower than those of the other two subgroups (*P* < 0.05 in Wilcoxon test). Using the corresponding values in the top 5% window for calculation of π values as the comparison node, the π values for China_Group1, China_Group2, and the Russia group were 0.00465, 0.00517, and 0.00526, respectively.

Tajima’s *D* analysis was used to examine positive selection effects by calculating differences in the number of segregating loci (θ_w_) and nucleotide diversity (π). In this study, Tajima’s *D* values were calculated using a 100 kb sliding window (Fig. 4d). The Russia group had the smallest Tajima’s *D* value, China_Group2 had the second-largest Tajima’s *D* value, and China_Group1 had the largest Tajima’s *D* value. In addition, 321, 119, and 49 genomic regions had a Tajima’s *D* value less than 0 in the Russia, China_Group2, and China_Group1 subgroups, respectively. In general, a genomic region with a Tajima’s *D* value less than 0 has a large number of low-frequency allelic loci, indicating that the region is under positive selection. The above-mentioned results, therefore, suggested that the Russia group included more rare alleles with more strongly positive selection than the two Chinese subgroups, which was consistent with the results of the LD analysis.

### Selective characterization of the genome of Chinese and Russian Apostichopus japonicus during geographic differentiation

**Fig. 5:**
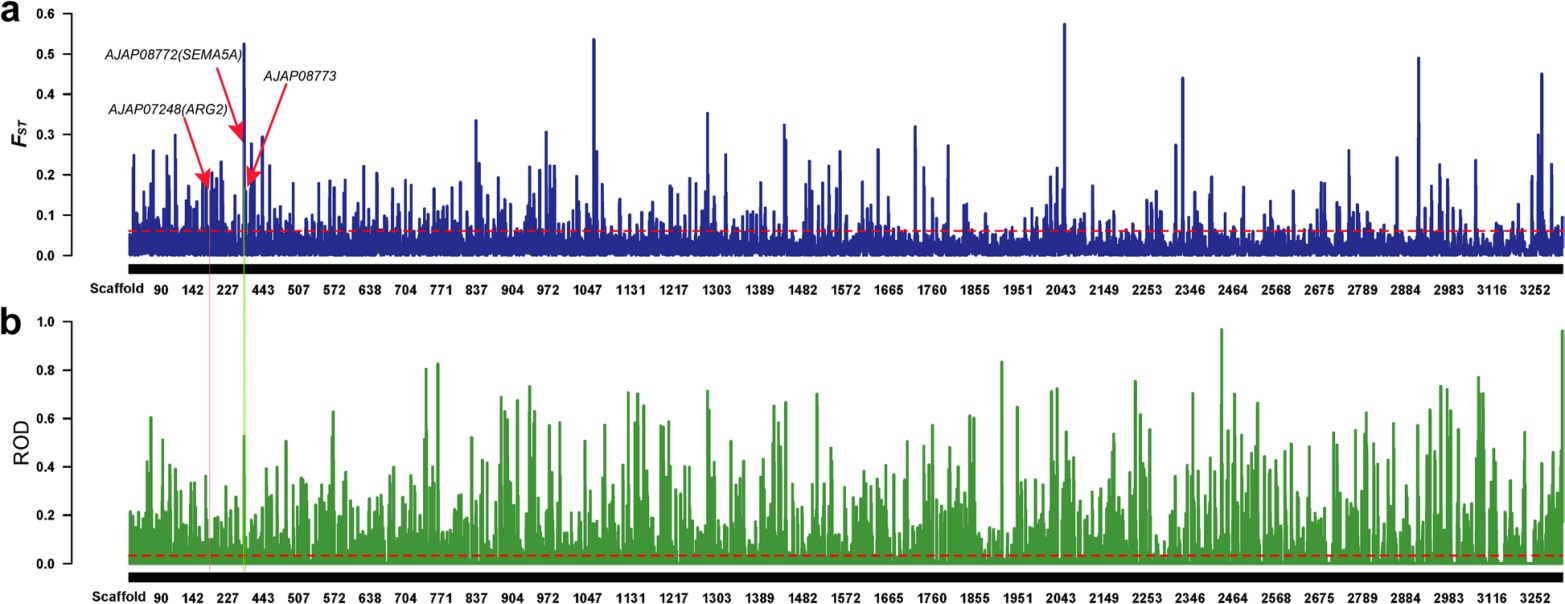
Population selection analysis of Chinese and Russian *A. japonicas*. **a** Based on the *F*_*ST*_ analysis of the inter-population divergence test, the threshold line is the value corresponding to the top 95% of *F*_*ST*_. **b** ROD analysis based on inter-group divergence test, the threshold line is the value corresponding to the top 95% of ROD

We used the *F*_*ST*_ calculation method proposed by Weir and Cockerham in 1984 [36]. The *F*_*ST*_ value of the top 5% was 0.061, and 2280 candidate regions were obtained using this threshold (Fig. 5a). The candidate regions covered a total of 1509 genes. The gene ontology (GO) terms that were significantly enriched among the candidate genes were mainly associated with immunity, and, notably, seven GO terms were associated with antimicrobial peptide or antimicrobial humoral response (Supplementary Fig. 3).

The ROD value of the top 5% was 0.068, and 3241 candidate regions were obtained using this threshold (Fig. 5b). A total of 1978 genes were covered by the candidate intervals. The significantly enriched GO terms among the candidate genes were mainly associated with inflammatory response (acute inflammatory response and cellular response to light stimulus) and cellular response to exogenous substances (cellular response to staurosporine, cell chemotaxis, cellular response to ammonium ion, and cellular response to light stimulus) (Supplementary Fig. 4). To increase the confidence of the differential intervals in the Chinese and Russian groups, we intersected the candidate intervals from *F*_ST_ and ROD values (Supplementary Fig. 5) to obtain 562 regions that covered 449 genes. The GO terms significantly enriched among the candidate genes were mainly associated with autophagosome and histone methyltransferase transporter (Supplementary Fig. 6).

**Fig. 6:**
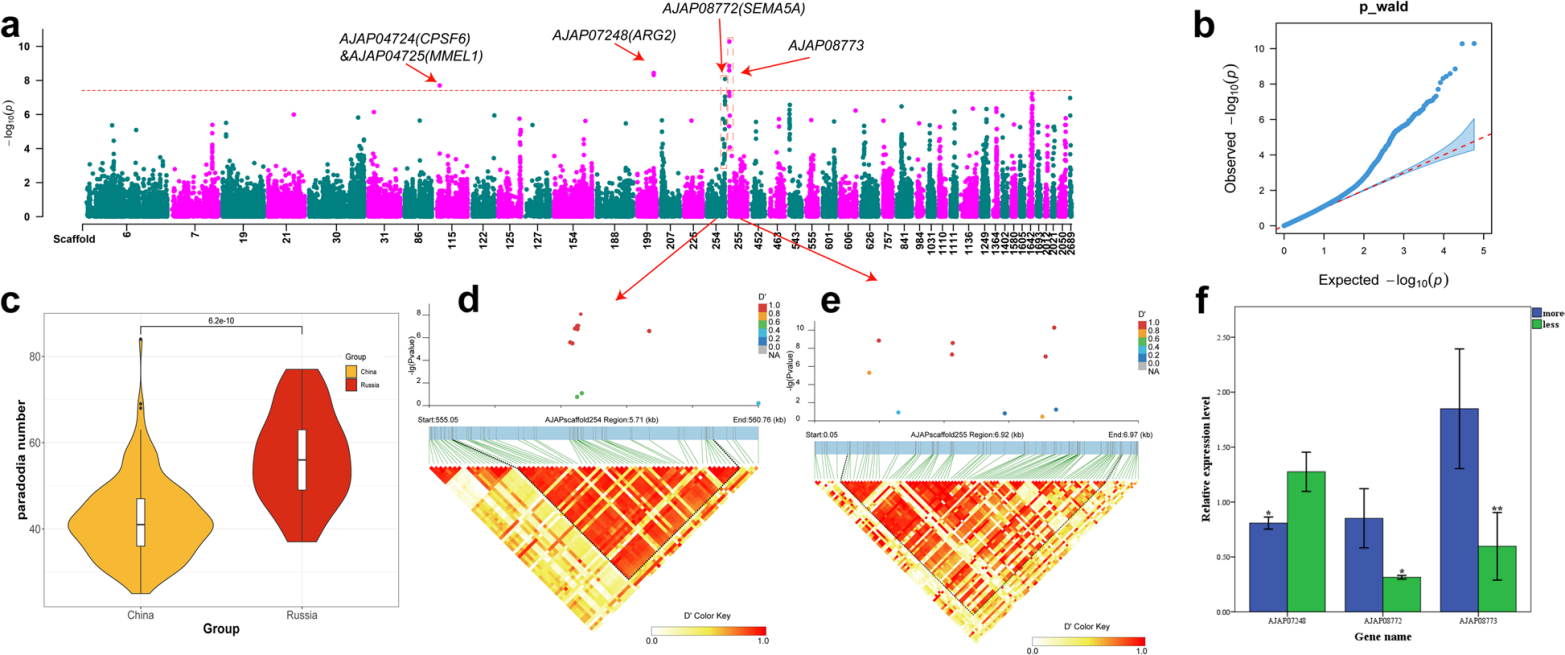
Genome-wide association analysis of parapodium number. **a** Manhattan chart display of GWAS results, Only the most significant 100 SNP loci located in Scaffold are shown in the figure. **b** QQ plot of GWAS results. **c** Comparative analysis of parapodium number between two subgroups using a t-test (**d**, **e**) Two LD blocks related to parapodium number. **f** Results of qRT-PCR validation of genes (** means *P *< 0.01 in t-test. * means *P* < 0.05 in t-test)

**Fig. 7:**
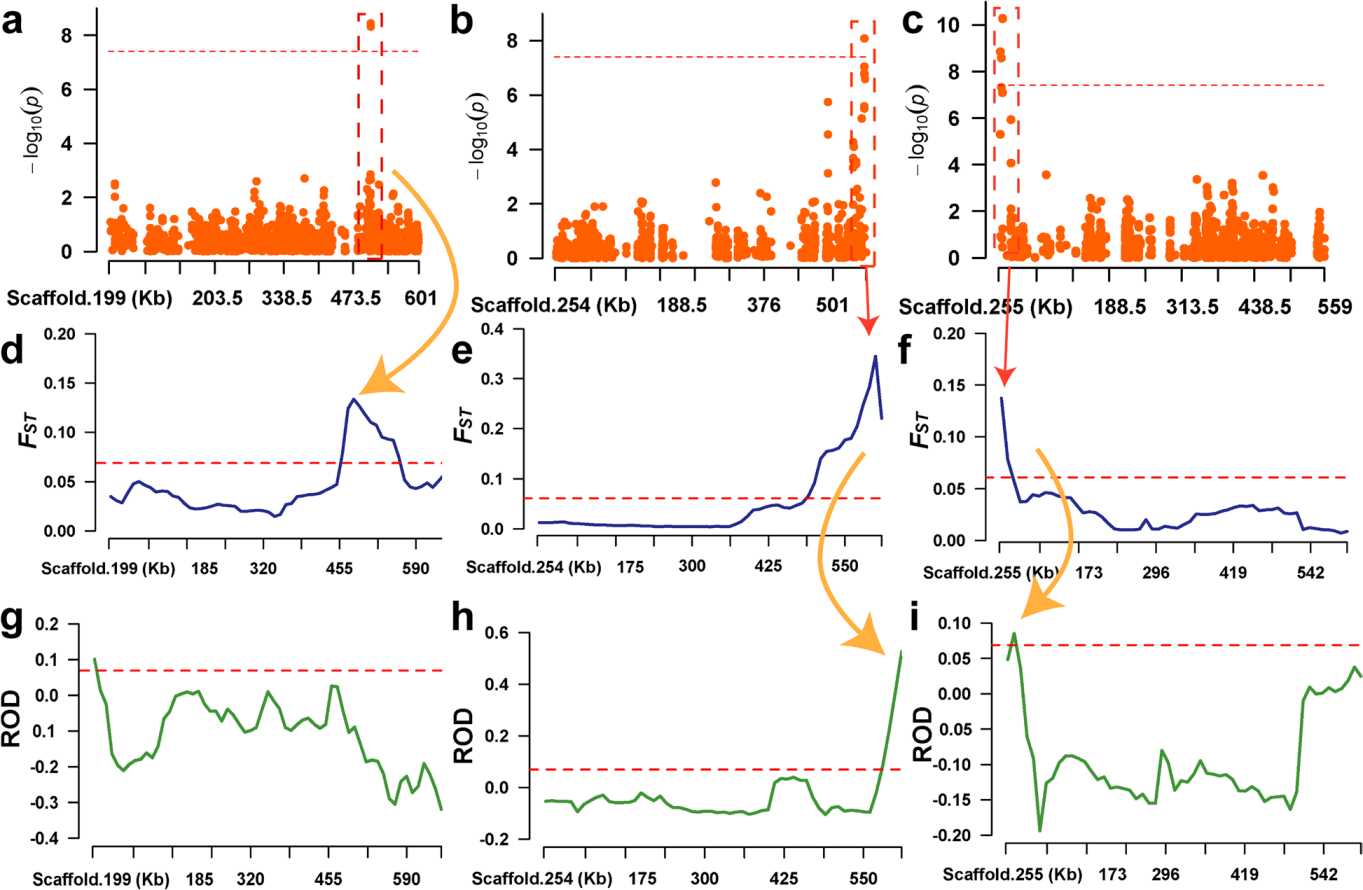
Parapodium number-associated SNP loci located in the selection interval

In this study, phenotypic differentiation in the number of parapodium was observed in the Russian and Chinese *A. japonicus* (Fig. 6c). The number of parapodium in the Russian group was significantly higher (mean 56.67) than that in the Chinese group (mean 41.83) (*P* < 0.05). To investigate the reasons for the difference in number of parapodium between the Russian and Chinese *A. japonicus*, we correlated the results of the GWAS analysis with those of the *F*_ST_ and ROD analyses (Fig. 7). Seven significantly associated SNP loci on Scaffold199, Scaffold254, and Scaffold255 were located within the selected *F*_ST_ interval (Fig. 7d–f), of which five were also located within the selected ROD interval (Fig. 7h, i). These results provide a genetic basis for the positive selection of the number of parapodium in Russian *A. japonicus*.

### Genome-wide association analysis of number of parapodium in Apostichopus japonicus

The parapodium – a hollow, conical, fleshy, spine-like tissue on the back of *A. japonicus* – is an important structure for respiration and exchange of information with the external environment. A linear mixed model was used for GWAS analysis, and eight SNP loci significantly associated with the number of parapodium were screened using a Bonferroni-corrected threshold (log_10_ *P* > 7.4; Fig. 6a). Five SNP loci were located in gene regions and three loci between genes, and these SNP loci were associated with a total of five genes in the genome (Table 3). The lower observed and expected values of the quantile–quantile plot conformed but the highest observed values deviated, indicating that the association model was reasonable and yielded reliable results (Fig. 6b). In the upstream and downstream linkage analysis of the SNPs, LD blocks were detected at Scaffold254 555 kb–560 kb and Scaffold255 0.05 kb–6.97 kb, which were located at AJAP08772 and AJAP08773, respectively (Fig. 6d, e). Gene homology annotations indicated that the gene AJAP08772 homolog *SEMA5A* encodes a protein with a Sema domain. The gene AJAP08773 encodes the glucose dehydrogenase C-terminus protein. In addition, three SNP-associated genes homologous to the annotated genes *CPSF6*, *MMEL1*, and *ARG2*, located on Scaffold115 and Scaffold199, encode a protein enabling exon–exon junction complex binding activity, membrane metallo-endopeptidase-like 1, and arginase, respectively. The quantitative real-time PCR validation of these five genes revealed that three genes were significantly differentially expressed (*P* < 0.05); AJAP08772 and AJAP08773 were significantly up-regulated in *A. japonicus* with a high number of parapodium (> 55) compared with individuals with a low number of parapodium (< 33), whereas the opposite was observed for AJAP07248 (Fig. 6f).

**Table 3:** SNP loci and candidate genes associated with the parapodium number traits of *A. japonicus*

Scaffold	Position(bp)	-logP	SNP	MAF	Gene	Gene symbol	Annotation
Scaffold115	109,061	7.70	A/T	0.07	AJAP04724; AJAP04725	*CPSF6*; *MMEL1*	exon-exon junction complex binding; Membrane metallo-endopeptidase-like 1
Scaffold199	507,705	8.32	G/A	0.22	AJAP07248	*ARG2*	Arginase
Scaffold199	507,706	8.44	T/A	0.22	AJAP07248	*ARG2*	Arginase
Scaffold254	557,682	8.08	G/A	0.24	AJAP08772	*SEMA5A*	Sema domain, seven thrombospondin repeats (Type 1 and type 1-like), transmembrane domain (TM) and short cytoplasmic domain, (Semaphorin)
Scaffold255	1422	8.85	T/C	0.16	AJAP08772; AJAP08773	*SEMA5A*; –-	Sema domain, seven thrombospondin repeats (Type 1 and type 1-like), transmembrane domain (TM) and short cytoplasmic domain, (Semaphorin); Glucose dehydrogenase C-terminus
Scaffold255	3001	8.58	C/T	0.13	AJAP08772; AJAP08773	*SEMA5A*; –-	Sema domain, seven thrombospondin repeats (Type 1 and type 1-like), transmembrane domain (TM) and short cytoplasmic domain, (Semaphorin); Glucose dehydrogenase C-terminus
Scaffold255	5172	10.28	T/G	0.14	AJAP08773	–-	Glucose dehydrogenase C-terminus
Scaffold255	5182	10.29	G/A	0.15	AJAP08773	–-	Glucose dehydrogenase C-terminus

## Discussion

### Analysis of the genetic structure and genetic differentiation of Russian and Chinese Apostichopus japonicus populations

The study found that individuals of the same species from different habitats exhibit trait divergence due to long-term adaptation to local environments, resulting in differences in their genetic backgrounds [37–39]. We believe that the population structure of the Chinese and Russian *A. japonicus* populations differed considerably, probably as a result of long-term adaptation to the local environment in terms of latitude, water temperature, light, and bait [40, 41]. Further analysis of the Chinese populations found that the HLW and LS populations showed a different population structure from the other four Chinese populations. *A. japonicus’*s reproductive behaviour is broadcast spawning, and ocean currents can affect aggregation and spawning [42]. The Bohai Sea is an inland sea [43], with a large number of freshwater rivers feeding into it, resulting in a lower salt content and density than the Yellow Sea [44]. Due to these reasons, we speculated that it was because the HLW and LS populations were located in the Bohai Sea, whereas the other populations were located in the Yellow Sea, and that different breeding environments caused genetic differentiation. In addition, the HLW and LS populations are of close geographic proximity and are prone to gene exchange. The results of the PCA and phylogenetic analysis were strongly consistent with the results of the admixture analysis.

Linkage disequilibrium analysis showed that the Russian population had a greater LD decay distance than the Chinese populations. A long LD decay distance indicates a high degree of genomic linkage and low genetic diversity, which may be subject to positive selection [45]. Tajima’s *D* test revealed a higher number of regions with values less than 0 for the Russia group than for the Chinese subgroups. Typically, genomic regions with Tajima’s *D* values less than 0 deviate from neutral selection, harbor reduced genetic diversity, and are subject to positive selection [46, 47]. Thus, these results suggested that the alleles of Russian *A. japonicus* has been subject to stronger positive selection. We hypothesize that this is because the Russian population has experienced a higher degree of natural selection to adapt to a high-latitude cold-water environment [48] and that its high-latitude location hinders the exchange of genes with low-latitude *A. japonicus*, resulting in easier retention of selected genes.

The nucleotide diversity (π) analysis of the three populations revealed that China_Group1 had the lowest genetic diversity and the Russia group had higher genetic diversity. In general, populations with high genetic diversity are usually subject to low levels of positive selection [49–51]. However, the genome of the Russia group, which had relatively high genetic diversity in this study, was subject to higher levels of selection and linkage, probably because the populations did not experience rigorous and systematic artificial selection, resulting in a genome with some linkage regions present but with less impact on overall genetic polymorphism.

### Selective characterization of the genome of Chinese and Russian A. japonicus during geographic differentiation

Functional enrichment analysis of candidate genes identified through population selection analysis revealed significant enrichment of pathways related to antimicrobial peptide, humoral immunity, and apoptosis in genes within candidate intervals determined through *F*_*ST*_ analysis. Seven of these pathways were associated with antimicrobial peptide or antimicrobial humoral response, which play a crucial role in immunity by disrupting bacterial membranes [52, 53] and activating the NFκB pathway to recruit antimicrobial peptides and pro-inflammatory cytokines to initiate defense mechanisms [54]. In addition, ROD analysis identified candidate genes with significantly increased likelihood of being associated with immunity, such as inflammatory response, in the GO terms, suggesting potential differences in immune function between Russian and Chinese groups of *A. japonicus*.

By combining the significantly different intervals of the China and Russia groups with the results of the GWAS analysis, we identified seven significantly associated SNP loci on Scaffold199, Scaffold254, and Scaffold255 located within the selected interval determined by the *F*_ST_ analysis, and five of these loci were also located within the selected interval obtained from the ROD analysis. These findings provide a genetic explanation for the positive selection of parapodium number in Russian *A. japonicus*.

### Genome-wide association analysis of number of parapodium in Chinese and Russian A. japonicus

In this study, eight SNP loci significantly associated with the number of parapodium were determined by the linear mixed model with Bonferroni correction. In the LD block analysis, LD blocks were identified at Scaffold254 555 kb–560 kb and Scaffold255 0.05 kb–6.97 kb, and the crucial genes AJAP08772 and AJAP08773 were identified in these two blocks associated with the number of parapodium in *A. japonicus*. SEMA5A, a homolog of AJAP08772, has been shown to stimulate endothelial cell proliferation and migration, while also inhibiting apoptosis [55]. The gene AJAP08773 encodes the protein glucose dehydrogenase C-terminus, which is involved in cell metabolism [56]. Gene expression analysis showed that the levels of AJAP08772 and AJAP08773 are significantly higher in individuals of the species *A. japonicus* with a larger number of parapodia. This suggests that these genes may play a role in enhancing the growth of parapodia in A. japonicus by increasing cell metabolism and proliferation rates.

In addition, we observed that the expression of AJAP07248 was significantly higher in the *A. japonicus* individuals that developed fewer parapodium. Gene homology annotation indicates AJAP07248 encodes arginase. Studies have shown that Nitric oxide (NO) regulation is the first step in the host defense mechanism of *A. japonicus* [57], that NO is a beneficial strategy for host cells to kill invasive pathogens [58]. Inhibition of arginase production leads to an increase in *A. japonicus* NO production and a enhance in the *A. japonicus*'s immune response to pathogenic microorganisms [59]. These results suggest that the number of parapodium in *A. japonicus* may be associated with immune-related functions, possibly through the regulation of arginase synthesis. Further research is needed to better understand the relationship between parapodium and immune function in this species.

## Conclusion

A total of 210 individuals of *Apostichopus japonicus* from China and Russia were subjected to high-throughput genome resequencing. The Chinese populations were divided into two subgroups that were primarily associated with geographic distribution. Genetic diversity analysis revealed a high degree of linkage between loci in the Russian population, which may have undergone stronger positive selection. Population selection analyses yielded genomic intervals between the Russia and China groups. Functional analysis of candidate genes within these intervals indicated differences in antimicrobial peptides, humoral immunity, apoptosis, and inflammatory responses. Three candidate genes significantly associated with parapodium number were identified by GWAS analysis. We hypothesized that AJAP08772 and AJAP08773 directly affect parapodium production by promoting endothelial cell proliferation and metabolism, whereas AJAP07248 indirectly affects parapodium production by participating in immune responses. The present study provides insight into the differences in genetic structure of Chinese and Russian populations of *A. japonicus*, and provides important information for subsequent genetic analysis and breeding of this species.

## Supplementary Information

**Additional file 1: Supplementary table 1.** Primer information of candidate genes.

**Additional file 2: Supplementary Fig. 1.** Location statistics of SNP loci.** Supplementary Fig. 2.** Density map of the number distribution of parapodia in 210 *Apostichopus japonicas.*** Supplementary Fig. 3.** GO analysis results of candidate genes obtained by* F*_*ST*_ analysis.** Supplementary Fig. 4.** GO analysis results of candidate genes obtained by ROD analysis.** Supplementary Fig. 5.** Intersection of candidate intervals for* F*_*ST*_ and ROD.** Supplementary Fig. 6.** Enrichment analysis of genes within *F*_*ST*_ and ROD candidate intervals.

## Data Availability

The original contributions presented in the study are included in the article/Supplementary Material. Further inquiries can be directed to the corresponding authors. The sequencing data that support the findings of this study are openly available in the NCBI Sequence Read Archive (SRA) under BioProject Accession No. PRJNA865892, and the datasets generated and/or analysed during the current study are available in the figshare repository, https://doi.org/10.6084/m9.figshare.20485317.v2.

## Abbreviations

BP: Biology process
*F*_*ST*_: Fixation index
GO: Gene ontology
GWAS: Genome-wide association study
InDel: Insertion-Deletion
LD: Linkage disequilibrium
LMM: Mixed linear model
PCA: Principal components analysis
qRT-PCR: Real-time quantitative PCR
ROD: The reduction of diversity
SNP: Single nucleotide polymorphism
π: Genetic diversity

## References

[1] Khotimchenko Y (2018). Pharmacological potential of sea cucumbers. Int J Mol Sci..

[2] Liu X, Sun Z, Zhang M, Meng X, Xia X, Yuan W, Xue F, Liu C (2012). Antioxidant and antihyperlipidemic activities of polysaccharides from sea cucumber Apostichopus japonicus. Carbohyd Polym.

[3] Oh G-W, Ko S-C, Lee DH, Heo S-J, Jung W-K (2017). Biological activities and biomedical potential of sea cucumber (Stichopus japonicus): a review. Fisheries Aquatic Sci.

[4] Ru X, Zhang L, Li X, Liu S, Yang H (2019). Development strategies for the sea cucumber industry in China. J Oceanol Limnol.

[5] Chang Y, Shi S, Zhao C, Han Z (2011). Characteristics of papillae in wild cultivated and hybrid sea cucumbers Apostichopus japonicus. Afr J Biotechnol..

[6] VandenSpiegel D, Flammang P, Fourmeau D, Jangoux M (1995). Fine structure of the dorsal papillae in the holothurioid Holothuria forskali (Echinodermata). Tissue Cell.

[7] Deichmann E (1956). Echinodermata. Vol. IV of The Invertebrates. The coelomate bilateria. Libbie Henrietta Hyman McGraw-Hill New York, 1955. vii + 763 pp. Illus. $10. Sci..

[8] Hoekstra LA, Moroz LL, Heyland A (2012). Novel Insights into the Echinoderm Nervous System from Histaminergic and FMRFaminergic-Like Cells in the Sea Cucumber Leptosynapta clarki. PLoS ONE.

[9] Yang H, Hamel JF, Mercier A. The sea cucumber Apostichopus japonicus: history, biology and aquaculture. Academic Press. 2015;9:1–23.

[10] Jin S, Su Y, Gao S, Wu F, Hu T, Liu J, Li W, Wang D, Chen S, Jiang Y (2018). Deep learning: individual maize segmentation from terrestrial lidar data using faster R-CNN and regional growth algorithms. Front Plant Sci.

[11] Varshney RK, Nayak SN, May GD, Jackson SA (2009). Next-generation sequencing technologies and their implications for crop genetics and breeding. Trends Biotechnol.

[12] Noemie VT, Sara M, Peter R, Maren W (2022). Unraveling the complex genetic basis of growth in New Zealand silver trevally Pseudocaranx georgianus. G3 (Bethesda).

[13] Jonathan SC, Beheregaray BL, Maren W (2022). Genomic prediction of growth in a commercially, recreationally, and culturally important marine resource, the Australian snapper (Chrysophrys auratus). G3 Bethesda..

[14] Zhu X, Ni P, Sturrock M, Wang Y, Ding J, Chang Y, Hu J, Bao Z (2022). Fine-mapping and association analysis of candidate genes for papilla number in sea cucumber, Apostichopus japonicus. Marine Life Sci Technol.

[15] Ge J, Tan J, Li F, Chen S, Liu C, Bian L (2020). A preliminary identification of genomic loci for body colour variation in the sea cucumber Apostichopus japonicus. Aquac Res.

[16] Guo C, Li Y, Xie J, Han L, Wang Y, Zhang X, Wu Y, Song J, Chang Y, Ding J. Revealing Selection in Breeding and Genetic Characteristics of Economically Important Traits of New Species of Apostichopus Japonicas Based on Genome Resequencing and GWAS Analysis. Front Marine Sci. 2022;9:948882.

[17] Zhixiong Z, Mei W, Junyi Y, Bo L, Leibin L, Yue S, Fei P, Peng X (2021). Genome-wide association analysis reveals genetic variations and candidate genes associated with growth-related traits and condition factor in Takifugu bimaculatus. Reprod Breed.

[18] Xin H, Fucun W, Haigang Q, Jie M, Wei W, Mingkun L, Li L, Guofan Z (2022). Whole-genome resequencing reveals the single nucleotide polymorphisms associated with shell shape in Crassostrea gigas. Aquaculture..

[19] Cue Z, Hui M, Liu Y, Song C, Li X, Li Y, Liu L, Shi G, Wang S, Li F (2015). High-density linkage mapping aided by transcriptomics documents ZW sex determination system in the Chinese mitten crab Eriocheir sinensis. Heredity..

[20] Li H, Durbin R (2010). Fast and accurate long-read alignment with burrows-wheeler transform. Bioinformatics.

[21] Zhang X, Sun L, Yuan J, Sun Y, Gao Y, Zhang L, Li S, Dai H, Hamel J-F, Liu C (2017). The sea cucumber genome provides insights into morphological evolution and visceral regeneration. PLoS Biol.

[22] McKenna A, Hanna M, Banks E, Sivachenko A, Cibulskis K, Kernytsky A, Garimella K, Altshuler D, Gabriel S, Daly M (2010). The genome analysis toolkit: a MapReduce framework for analyzing next-generation DNA sequencing data. Genome Res.

[23] Cingolani P, Platts A, Wang LL, Coon M, Nguyen T, Wang L, Land SJ, Lu X, Ruden DM (2012). A program for annotating and predicting the effects of single nucleotide polymorphisms SnpEff. Fly.

[24] Yang J, Lee SH, Goddard ME, Visscher PM, Gondro C, van der Werf J, Hayes B (2013). Genome-Wide Complex Trait Analysis (GCTA): Methods, Data Analyses, and Interpretations. Genome-Wide Association Studies and Genomic Prediction.

[25] Alexander DH, Novembre J, Lange K (2009). Fast model-based estimation of ancestry in unrelated individuals. Genome Res.

[26] Purcell S, Neale B, Todd-Brown K, Thomas L, Ferreira MAR, Bender D, Maller J, Sklar P, De Bakker PIW, Daly MJ (2007). PLINK: a tool set for whole-genome association and population-based linkage analyses. Ame J Hum Gen.

[27] Zhang C, Dong S-S, Xu J-Y, He W-M, Yang T-L (2019). PopLDdecay: a fast and effective tool for linkage disequilibrium decay analysis based on variant call format files. Bioinformatics.

[28] Danecek P, Auton A, Abecasis G, Albers CA, Banks E, DePristo MA, Handsaker RE, Lunter G, Marth GT, Sherry ST (2011). The variant call format and VCFtools. Bioinformatics.

[29] Li Y, Cao K, Zhu G, Fang W, Chen C, Wang X, Zhao P, Guo J, Ding T, Guan L (2019). Genomic analyses of an extensive collection of wild and cultivated accessions provide new insights into peach breeding history. Genome Biol.

[30] Cantalapiedra CP, Hernández-Plaza A, Letunic I, Bork P, Huerta-Cepas J (2021). eggNOG-mapper v2: functional annotation, orthology assignments, and domain prediction at the metagenomic scale. Mol Biol Evol.

[31] Carlson M, Pagès H. AnnotationForge: tools for building SQLite-based annotation data packages. R package version 1.34.0. 2021.

[32] Wu T, Hu E, Xu S, Chen M, Guo P, Dai Z, Feng T, Zhou L, Tang W, Zhan L (2021). Cluster profiler 4.0: A universal enrichment tool for interpreting omics data. Innov..

[33] Zhou X, Stephens M (2012). Genome-wide efficient mixed-model analysis for association studies. Nat Genet.

[34] Yang J, Zeng J, Goddard ME, Wray NR, Visscher PM (2017). Concepts, estimation and interpretation of SNP-based heritability. Nat Genet.

[35] Cardellino R, Rovira J. Mejoramiento genético animal. Hemisferio Sur. 1987:253.

[36] Weir BS, Cockerham CC (1984). Estimating F-statistics for the analysis of population structure. Evol..

[37] Daishi Y, Satoshi C (2021). Comparing the genetic diversity and population structure of sister marine snails having contrasting habitat specificity. Mol Biol Rep..

[38] Yanglei J, Xiao L (2022). Diversification of the aquaporin family in geographical isolated oyster species promote the adaptability to dynamic environments. BMC genomics..

[39] Waples RS, Ford MJ, Nichols K, Kardos M, Myers J, Thompson TQ, Anderson EC, Koch IJ, McKinney G, Miller MR et al. Implications of Large-Effect Loci for Conservation: A Review and Case Study with Pacific Salmon. J Heredity. 2022;113(2):121–44. 10.1093/jhered/esab069 35575083

[40] Dvoretsky AG, Dvoretsky VG. Cucumaria in Russian waters of the Barents Sea: Biological aspects and aquaculture potential. Front Marine Sci. 2021;8:613453.

[41] Hu M, Li Q, Li L (2010). Effect of salinity and temperature on salinity tolerance of the sea cucumber Apostichopus japonicus. Fish Sci.

[42] Marquet N, Hubbard PC, da Silva JP, Afonso J, Canário AVM (2018). Chemicals released by male sea cucumber mediate aggregation and spawning behaviours. Sci Rep.

[43] Zhang Z, Zhu M, Wang Z, Wang J (2006). Monitoring and managing pollution load in Bohai Sea PR China. Ocean Coastal Management.

[44] Yu H, Bao X, Lu CL, Chen X, Kuang L (2009). Analyses of the long-term salinity variability in the Bohai Sea and the northern Huanghai (Yellow) Sea. Acta Oceanol Sin.

[45] Blair MW, Cortés AJ, Farmer AD, Huang W, Ambachew D, Penmetsa RV, Carrasquilla-Garcia N, Assefa T, Cannon SB. Uneven recombination rate and linkage disequilibrium across a reference SNP map for common bean (Phaseolus vulgaris L.). PLOS ONE. 2018;13(3):e0189597. 10.1371/journal.pone.0189597 PMC5844515 29522524

[46] TeYu L, PeiLuen L, YuanHuan Y, WenChien H, JenChieh S, HungDu L, WeiCheng J, TakKei C, Fan L (2021). Amphidromous but endemic: Population connectivity of Rhinogobius gigas (Teleostei: Gobioidei). PloS one.

[47] Rashid S, Jan H, Tania M, Aniqa E, Fraz A, Saeeda Z (2021). Detection of whole genome selection signatures of Pakistani Teddy goat. Mol Biol Rep..

[48] Jørgensen LL, Logerwell EA, Strelkova N, Zakharov D, Roy V, Nozères C, Bluhm BA, Hilma Ólafsdóttir S, Burgos JM, Sørensen J (2022). International megabenthic long-term monitoring of a changing arctic ecosystem: Baseline results. Prog Oceanogr.

[49] Concetta B, Angélique B, Sylvain G, Jacques D, Nancy T, Monique D, David P (2021). The road to Sorghum domestication: evidence from nucleotide diversity and gene expression patterns. Front Plant Sci..

[50] Benoit L, Verônica T, Carolina S, Sonia A, Magdalena R, Rubens PJ (2021). Extremely low nucleotide diversity among thirty-six new chloroplast genome sequences from Aldama (Heliantheae, Asteraceae) and comparative chloroplast genomics analyses with closely related genera. PeerJ..

[51] Magnus AR, Anna A, Ulrika L, Páll Ó, Stefania G, Björn N, Anders B (2021). Genomic characterization of the barnacle Balanus improvisus reveals extreme nucleotide diversity in coding regions. Marine Biotechnol (New York, NY)..

[52] Shai Y (1995). Molecular recognition between membrane-spanning polypeptides. Trends Biochem Sci..

[53] Epand RM, Shai Y, Segrest JP, Anantharamiah GM: Mechanisms for the modulation of membrane bilayer properties by amphipathic helical peptides. Biopolymers. 1995;37(5):319–38. 10.1002/bip.360370504 7632881

[54] Liyanage DS, Omeka WKM, Nadarajapillai K, Lim C, Yang H, Choi JY, Kim KM, Noh JK, Jeong T, Lee J. Molecular cloning, expression analysis of interleukin 17D (cysteine knot cytokine) from Amphiprion clarkii and their functional characterization and NFκB pathway activation using FHM cells. Fish Shellfish Immunol. 2022;126:217–26. 10.1016/j.fsi.2022.05.047 35636699

[55] Sadanandam A, Rosenbaugh EG, Singh S, Varney M, Singh RK (2009). Semaphorin 5A promotes angiogenesis by increasing endothelial cell proliferation, migration, and decreasing apoptosis. Microvasc Res..

[56] Xiaojun Z, Lina S, Jianbo Y, Yamin S, Yi G, Libin Z, Shihao L, Hui D, Jean-François H, Chengzhang L (2017). The sea cucumber genome provides insights into morphological evolution and visceral regeneration. PLoS Biol..

[57] Yina S, Chenghua L, Weiwei Z, Zhenhui W, Zhimeng L (2016). The first description of complete invertebrate arginine metabolism pathways implies dose-dependent pathogen regulation in Apostichopus japonicus. Sci Rep.

[58] Das P, Lahiri A, Lahiri A, Chakravortty D (2010). Modulation of the Arginase Pathway in the Context of Microbial Pathogenesis: A Metabolic Enzyme Moonlighting as an Immune Modulator. PLoS Pathog.

[59] Shao Y, Li C, Zhang W, Xu W, Duan X, Li Y, Qiu Q, Jin C (2016). Cloning and comparative analysis the proximal promoter activities of arginase and agmatinase genes in Apostichopus japonicus. Dev Comp Immunol.

