# Supplementary material for: Whole-genome resequencing reveals genetic differences and the genetic basis of parapodium number in Russian and Chinese Apostichopus japonicus
Source: BMC Genomics. 2023 Jan 16;24:25. doi: 10.1186/s12864-023-09113-x (PMC9843871; doi:10.1186/s12864-023-09113-x)
Supplement: Supplementary file 1 — Additional file 1: Supplementary table 1. Primer information of candidate genes. [file 12864_2023_9113_MOESM1_ESM.docx]

Supplementary table 1 Primer information of candidate genes

| Gene name | Forward primer | Reverse primer |
| --- | --- | --- |
| AJAP07248 | 5' TAATAGAGTGGATGGTGGCTTCAG 3' | 5' GTCTGACCTCTACGACCCGAAC 3' |
| AJAP08772 | 5' ACCCTCCACCAGAACAAATGAAAAT 3' | 5' CGGAATGGGCACACACTGTAGAAAC 3' |
| AJAP08773 | 5' CTGGAAGTGACAGTTGAGGGAC 3' | 5' TTTCATTTGTTCTGGTGGAGGG 3' |
