# Supplementary material for: Whole-genome resequencing reveals genetic differences and the genetic basis of parapodium number in Russian and Chinese Apostichopus japonicus
Source: BMC Genomics. 2023 Jan 16;24:25. doi: 10.1186/s12864-023-09113-x (PMC9843871; doi:10.1186/s12864-023-09113-x)
Supplement: Supplementary file 2 — Additional file 2: Supplementary Fig. 1. Location statistics of SNP loci. Supplementary Fig. 2. Density map of the number distribution of parapodia in 210 Apostichopus japonicas. Supplementary Fig. 3. GO analysis results of candidate genes obtained by FST analysis. Supplementary Fig. 4. GO analysis results of candidate genes obtained by ROD analysis. Supplementary Fig. 5. Intersection of candidate intervals for FST and ROD. Supplementary Fig. 6. Enrichment analysis of genes within FST and ROD candidate intervals. [file 12864_2023_9113_MOESM2_ESM.docx]

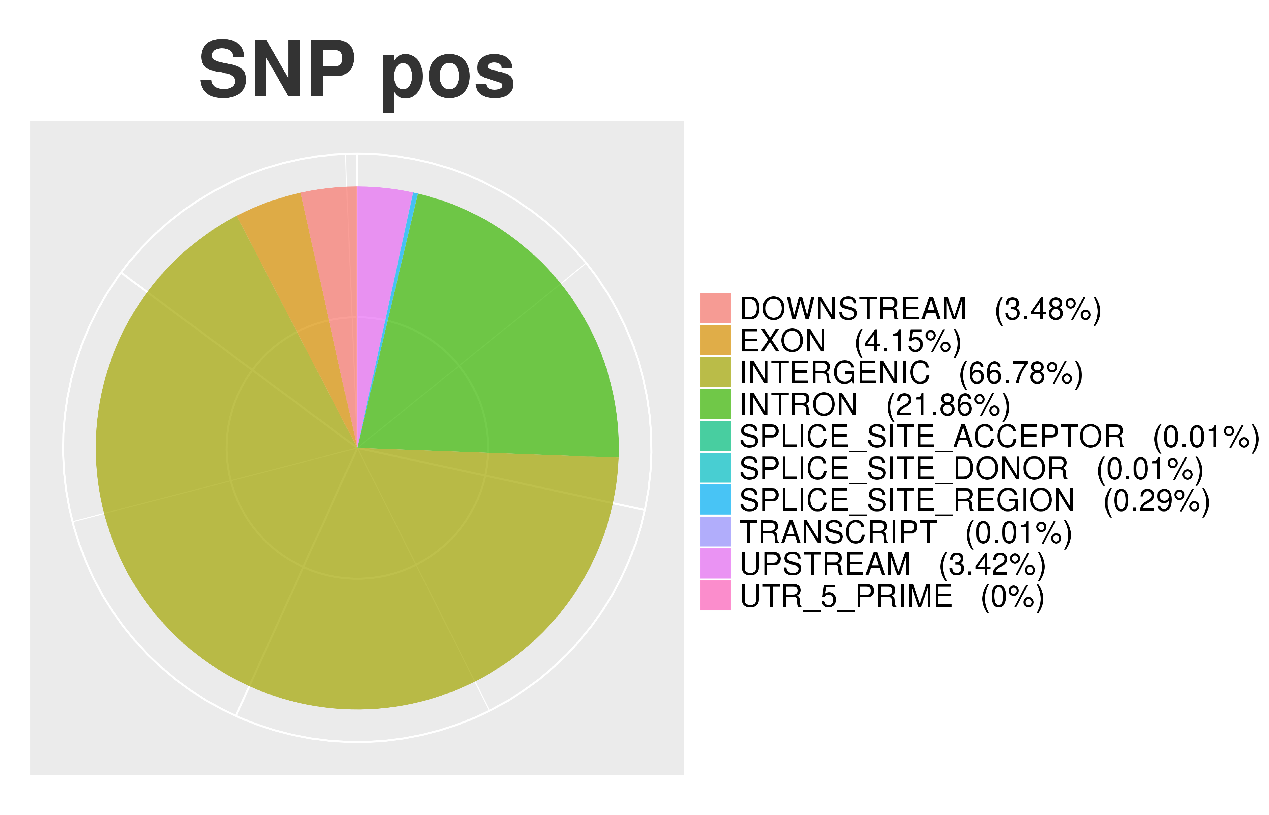


Supplementary Fig. 1 Location statistics of SNP loci


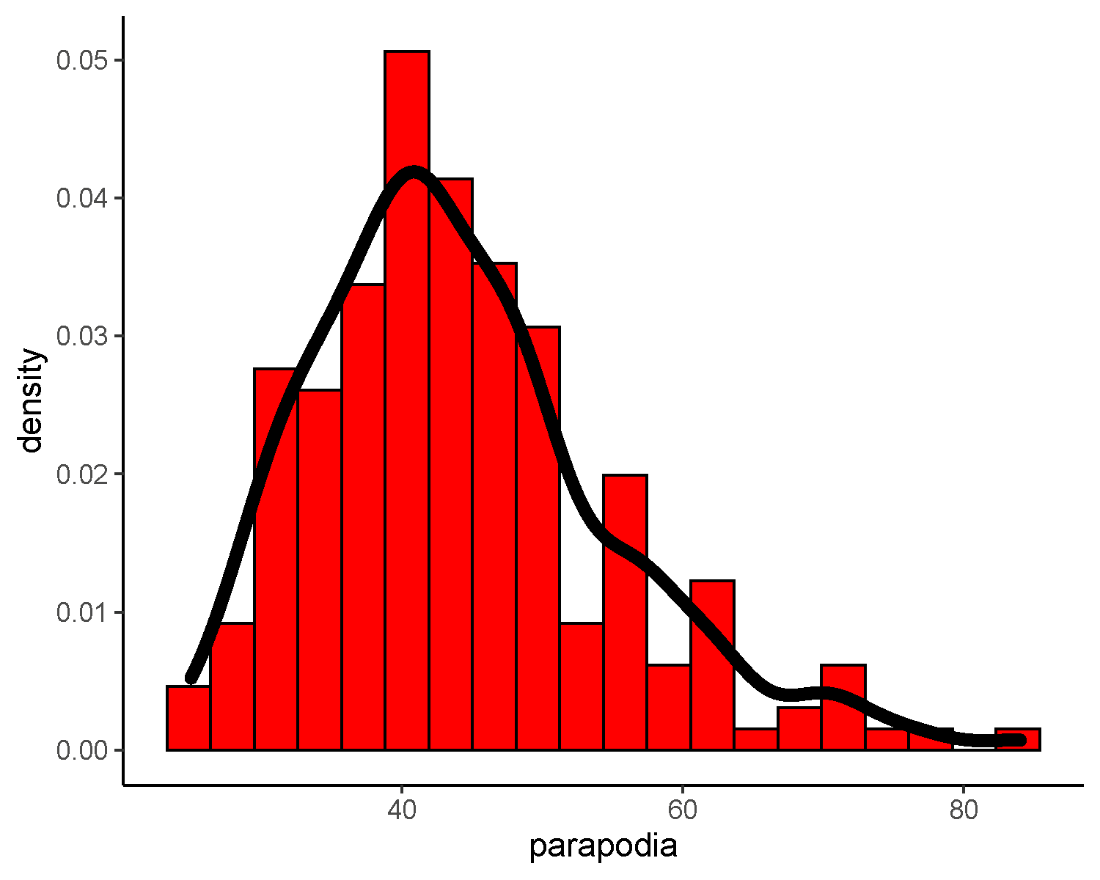


Supplementary Fig. 2 Density map of the number distribution of parapodia in 210 *Apostichopus japonicus*
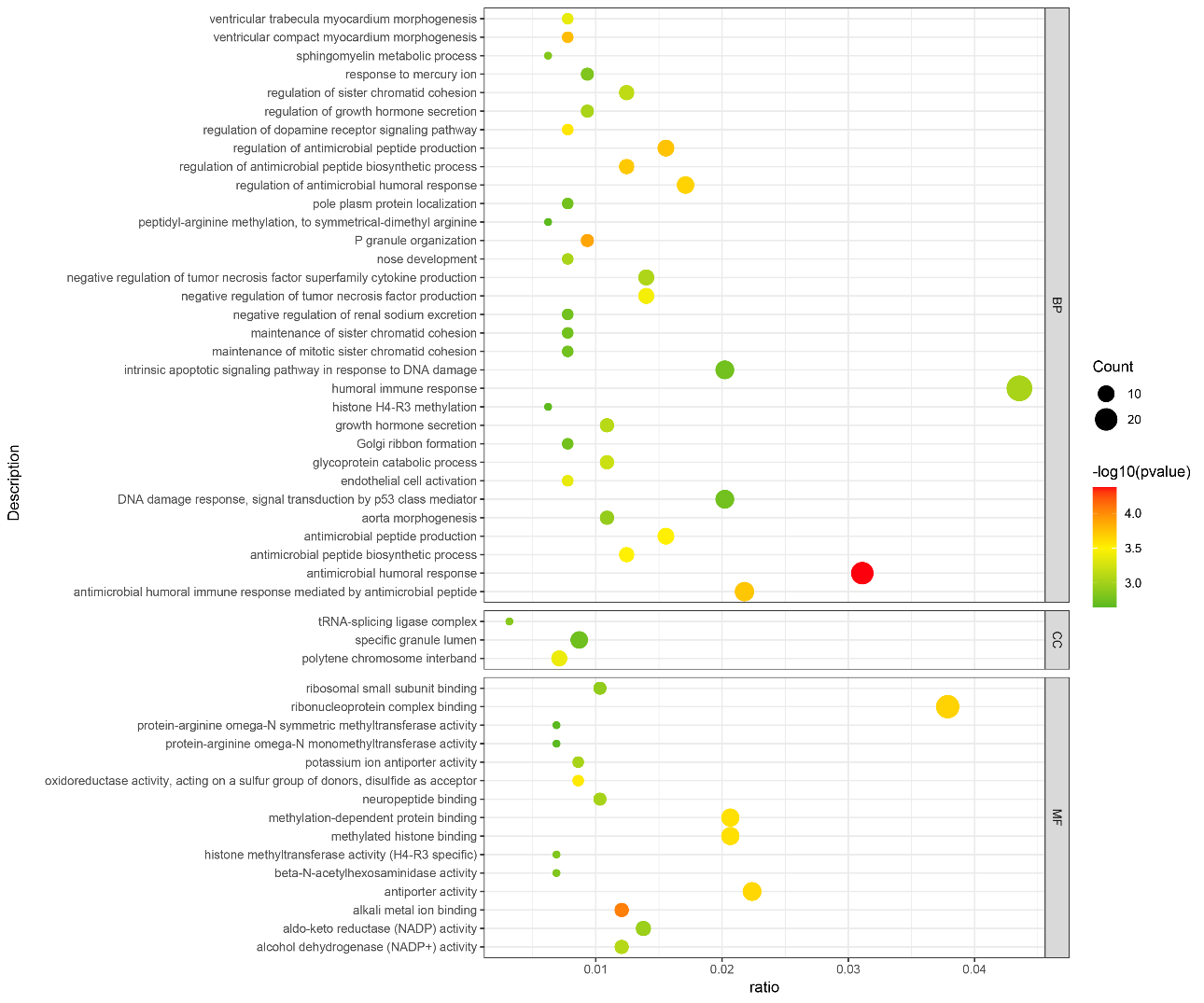


Supplementary Fig. 3 GO analysis results of candidate genes obtained by *F_ST_* analysis


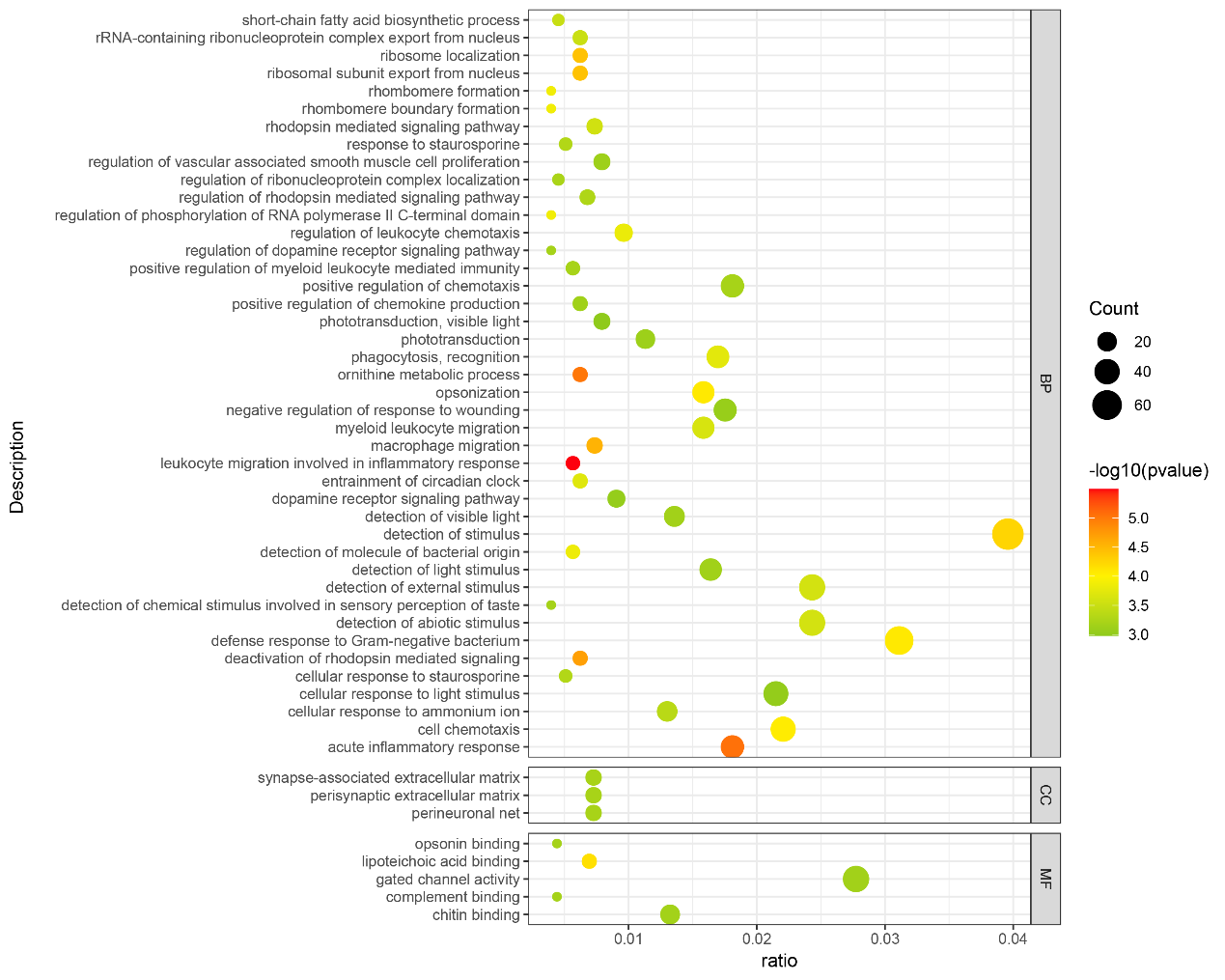


Supplementary Fig. 4 GO analysis results of candidate genes obtained by ROD analysis


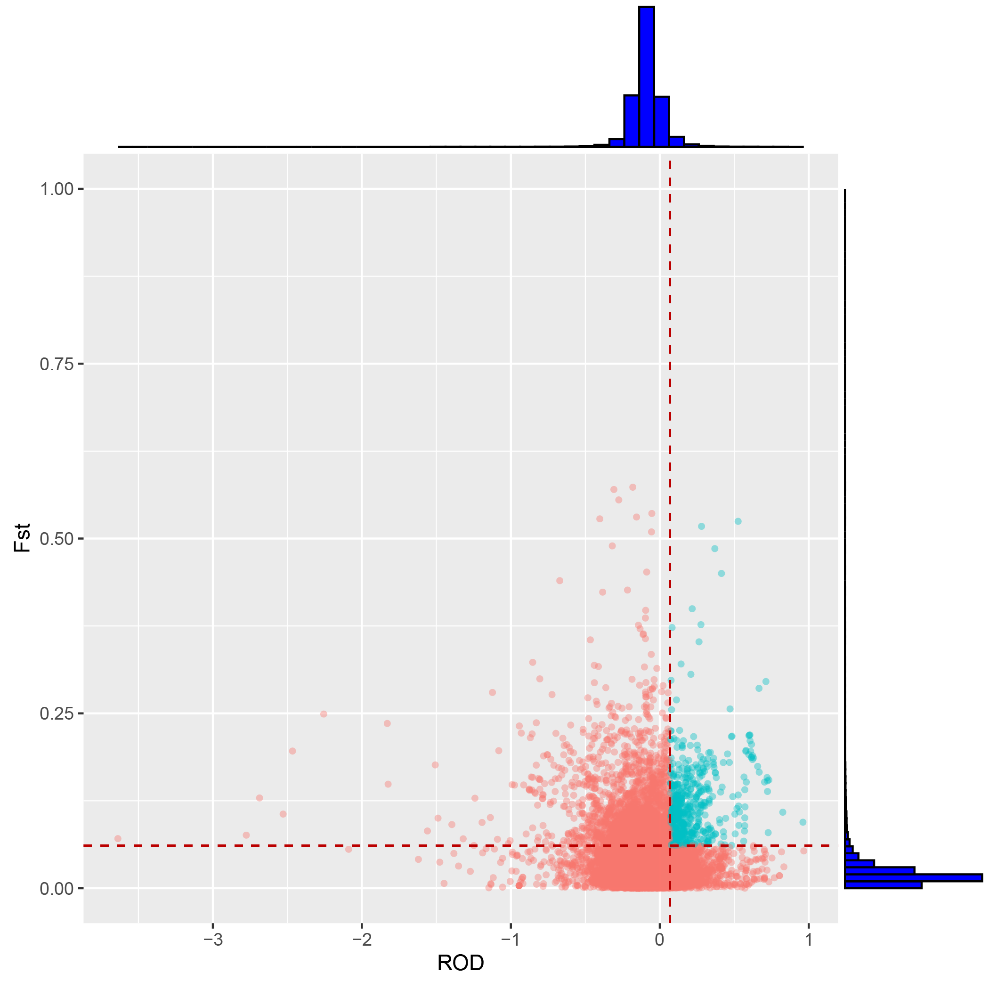


Supplementary Fig. 5 Intersection of candidate intervals for *F_ST_* and ROD


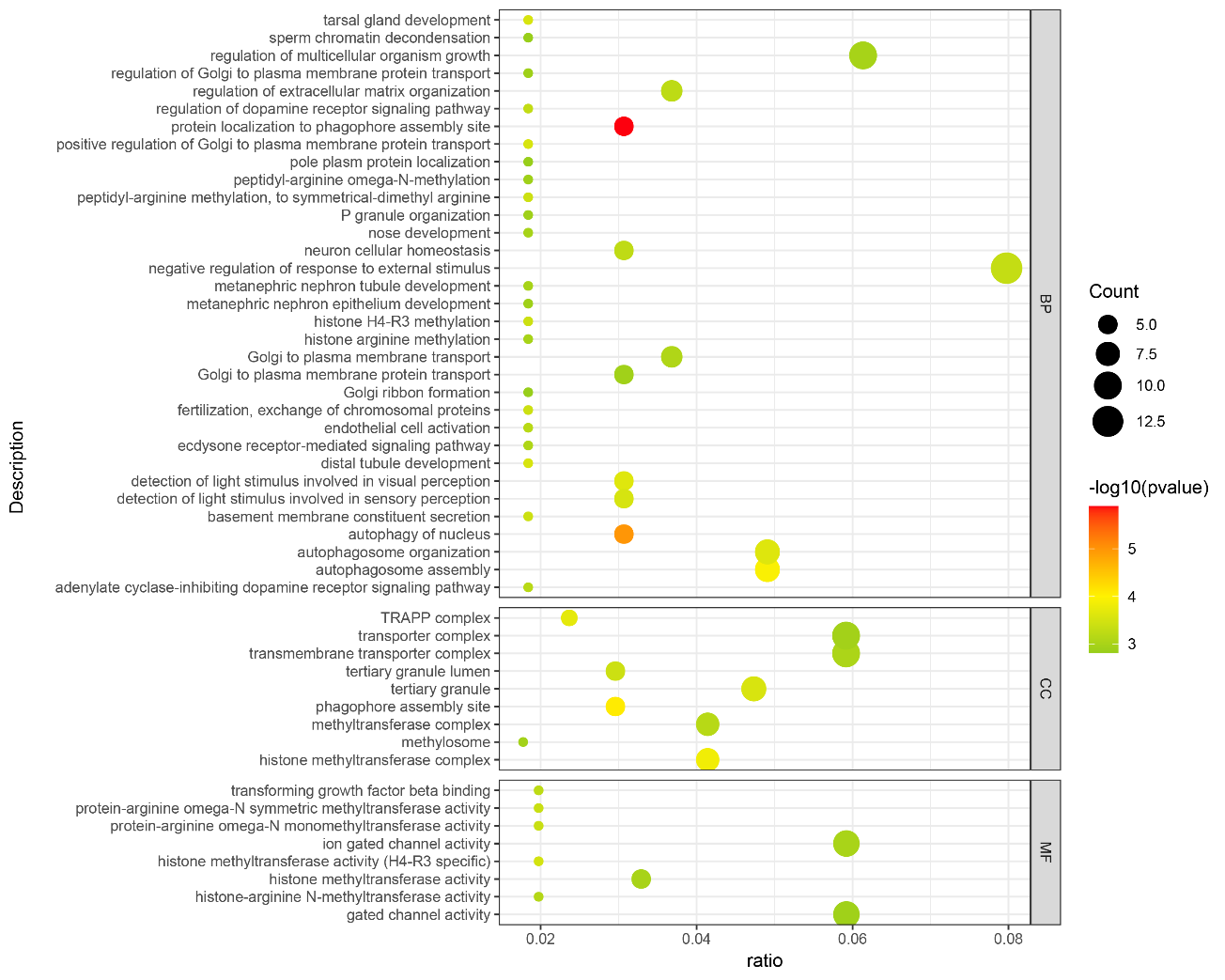


Supplementary Fig. 6 Enrichment analysis of genes within *F_ST_* and ROD candidate intervals
